# Preparing for Winter: The Transcriptomic Response Associated with Different Day Lengths in *Drosophila montana*

**DOI:** 10.1534/g3.116.027870

**Published:** 2016-03-11

**Authors:** Darren J. Parker, Michael G. Ritchie, Maaria Kankare

**Affiliations:** * Department of Biological and Environmental Science, University of Jyväskylä, FI-40014, Finland; † Centre for Biological Diversity, School of Biology, University of St Andrews, Fife, KY16 9TH, UK

**Keywords:** photoperiod, transcriptomics, overwintering, gene expression, diapause

## Abstract

At northern latitudes, the most robust cue for assessing the onset of winter is the shortening of day lengths. Many species use day length as a cue to increase their cold tolerance and/or enter into diapause, but little is known about changes in gene expression that occur under different day lengths. We investigate the gene expression changes associated with differences in light/dark cycles in *Drosophila montana*, a northerly distributed species with a strong adult photoperiodic reproductive diapause. To examine gene expression changes induced by light both prior to and during diapause, we used both nondiapausing and diapausing flies. We found that the majority of genes that are differentially expressed between different day lengths in nondiapausing and diapausing flies differ. However, the biological processes involved were broadly similar. These included neuron development and metabolism, which are largely consistent with an increase in cold tolerance previously observed to occur in these flies. We also found that many genes associated with reproduction change in expression level between different day lengths, suggesting that *D. montana* use changes in day length to cue changes in reproduction both before and after entering into diapause. Finally, we also identified several interesting candidate genes for light-induced changes including *Lsp2*, *para*, and *Ih*.

Seasonal shifts in climatic conditions pose numerous problems for ectothermic organisms, especially those living at high latitudes, and as a result they have evolved several adaptations to deal with these (Salt 1961; Weiser 1970; Lee and Costanzo 1998; Lee 2010). Many of the adaptations, for example increased cold tolerance and entering into diapause, are facultatively controlled and cued by the changing environmental conditions, in particular, differences in day length and average temperature (Weiser 1970; Denlinger 2002; Lee 2010). The ability to enter facultative diapause with the onset of winter is widespread across northern adapted species, and has been extensively documented as an overwintering strategy in insects and plants (Andrewartha 1952; Perry 1971; Tauber *et al.* 1985). The induction of diapause is often influenced by photoperiod, as it is a reliable cue to the onset of winter (Weiser 1970; Saunders 2008, 2012). In insects, research has focused on how day length is perceived and how long is needed for diapause to be induced. Species typically have a narrow “critical day length” (CDL), the day length where half of the individuals of a specific population enter diapause. Since the CDL is relatively short in comparison to the variation in day lengths normally experienced by a particular species, it is also likely that individuals prepare for seasonal changes by adjusting their physiology using changes in day length that are significantly shorter or longer than the CDL, both before and after diapause has been induced (Denlinger 2002).

Photoperiodic induction of diapause clearly involves the interaction of several distinct mechanisms including: photoreception, measurement of day length, accumulation of “diapause-inducing” day lengths, and downstream output pathways, triggered when a certain threshold is reached regulating the transition into diapause (Emerson *et al.* 2009; Saunders 2011, 2012). The genetic basis of each of these stages has been investigated in several orders of insect and there is a great deal of diversity (Denlinger 2002). This reflects the independent evolution of facultative diapause in many insect groups as they extended their range into higher latitudes (Saunders 2011). Despite this, most studies implicate the circadian clock as the central mechanism for the measurement of day length and thus the induction of diapause (Denlinger 2002; Saunders 2011). In contrast, genes involved in downstream output pathways of diapause are more diverse between species, which perhaps reflects independent origins of diapause, physiological responses, and/or different selective histories of different species (see the following for reviews: Denlinger 2002; Saunders 2011).

The goal of this study was to examine the gene expression changes that occur in response to the seasonal cue of differing day lengths in *Drosophila montana*, a northern malt fly species from the *Drosophila virilis* group. Females of this species enter reproductive diapause at the end of the summer, overwinter as adults, and develop mature ovaries prior to mating and reproduction the following spring. The developmental pathways leading to reproductive (nondiapausing) or diapausing states have been shown to be largely cued by changes in day length (Lumme 1978). In the wild, the majority of the females that emerge before the critical day length will develop mature ovaries, while the ones that emerge after this day length has been reached will enter diapause. Those females that develop mature ovaries will produce progeny during the spring/summer, while the diapausing females will overwinter as adults and produce progeny the following spring/summer (Lumme 1978). To examine gene expression changes during these processes, we chose three different light:dark (LD) cycles representing important time points in *D. montana*’s life: summer (LD = 22:2, beginning of July, when flies are actively reproducing), CDL (LD = 18.5:5.5, end of July, see Tyukmaeva *et al.* 2011), and late summer (LD = 16:8, mid-August, when practically all flies have entered diapause and are preparing to overwinter) (Figure 1). Note, the CDL is known to be population-specific and these values are correct for the Northern Finnish population of *D. montana* used in this study. By using the CDL as one of our conditions, we were able to collect both diapausing and nondiapausing flies from the same LD cycle. These samples could then be used to examine gene expression changes between (nondiapausing) flies kept in light conditions typical of summer and CDL LD cycles, and between (diapausing) flies kept in CDL and late summer LD cycles. This allows us to avoid the problems associated with comparing gene expression of heterogeneous tissues which would arise from any direct comparison between diapausing and nondiapausing samples (Neville and Goodwin 2012), which differ drastically in ovary size (Salminen *et al.* 2012).

**Figure 1:**
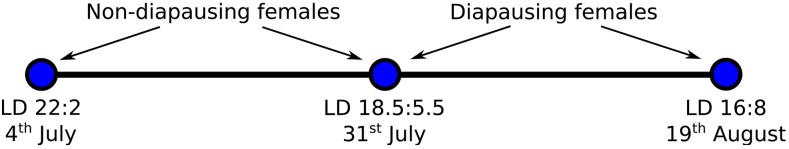
Light:dark (LD) cycles used in this study and the dates they correspond to in the *D. montana* population where the flies originate in nature (Oulanka, Finland, 66.22°N).

Overall, this strategy enabled us to identify genes associated with different day lengths in both diapausing and nondiapausing females. Previous work on photoperiodic diapause in many species has implicated circadian genes as important for the induction of diapause (Denlinger 2002; Saunders 2011), and we hypothesized that these genes may also be involved in cueing changes in response to day length changes more generally. Previous work has also shown that cold tolerance increases with shortening day length in both diapausing and nondiapausing *D. montana* (Vesala *et al.* 2012a), suggesting that shortening day length cues transcription of genes involved in increasing cold tolerance. Since the increase in cold tolerance is similar in both diapausing and nondiapausing flies (Vesala *et al.* 2012a), we also hypothesized that the genes underlying this change would show similar expression changes in both types of fly. We examined these expectations by identifying which genes, and the processes they are associated with, show similar or contrasting changes in gene expression in response to different (shorter) day lengths in both diapausing and nondiapausing flies.

## Materials and Methods

### Flies

Flies used were taken from three *D. montana* isofemale lines (175OJ8, 3OL8, and 265OJ8) established from the progenies of wild-caught fertilized females from Oulanka, Finland (66.22°N) in 2008. The lines have been maintained since their establishment on malt food in continuous light at 19 ± 1°, 65% humidity. Females used in this experiment were collected in 2010 within 1 d of eclosion using light CO_2_ anesthesia and transferred into a climate chamber (Sanyo MLR-351H) for 21 d at one of the three light-dark (LD) cycles: 22:2, 18.5:5.5, or 16:8. Thus, we had four experimental groups of flies: nondiapausing flies from 22:2 LD, both diapausing and nondiapausing flies from 18.5:5.5 LD, and diapausing females from 16:8 LD. The reproductive state of females was determined by examining their ovarian development via dissection under a light microscope. Previtellogenic small and transparent ovaries with no yolk accumulation or visible segments were classified as diapausing, and large vitellogenic ovaries with visible eggs as nondiapausing (Tyukmaeva *et al.* 2011). Flies that had intermediate ovaries with some yolk accumulation and segments visible but no eggs were not used.

### RNA extraction and sequencing

Female flies for RNA extraction were flash frozen in liquid nitrogen 5 hr after lights on in the chambers (Zeitgeber = 5) and submerged into RNAlater ICE solution (Ambion). Flies were pooled into 12 samples (three from each of the experimental groups), with 10 whole flies pooled in each sample.

RNA was extracted from each sample using Tri Reagent (Sigma-Aldrich) followed by RNeasy Mini kit (Qiagen) purification with DNase treatment. Purity of the RNA was checked using a NanoDrop ND-1000 spectrophotometer (NanoDrop Technologies) and integrity with a 2100 Bioanalyzer (Agilent Technologies). Extracted RNA was sequenced at the Turku Centre for Biotechnology (Turku, Finland) using the SOLiD 5500 XL platform to produce 69 million 75 + 35 bp paired end reads. Raw sequence reads were trimmed using SOLiD TRIM (with run options: -p 3 -q 22 -y y -e 2 -d 10) to remove polyclonal errors from the data (Sasson and Michael 2010). The reads that passed this filter were then error corrected using SOLiD Accuracy Enhancer Tools (SAET) to reduce the amount of color calling errors, or erroneous bases, in the sequence. Remaining low quality bases at the end of the reads were then trimmed using CLC Genomics Workbench 5.0.1 (CLC) (quality score: 0.02).

### Assembly and annotation

As *D. montana* has no reference genome currently available, we produced a *de novo* assembly using CLC Bio (default options, minimum contig size = 200 bp) on the reads obtained here, plus others from an acclimation study (Parker *et al.* 2015) and a diapause study (M. Kankare *et al.*, unpublished data) to produce the reference transcriptome, consisting of 31,880 contigs (N50 = 527). Contigs from the *de novo* assembly were annotated using Blast2GO (Conesa *et al.* 2005) by blasting (blastX) contigs to the nonredundant protein collection (nr) database. Contigs that did not obtain a significant blast hit (E-value > 0.001) from this were blasted (blastN) to the nonredundant nucleotide collection (nt) database. Contigs still without a hit following this were blasted (blastN) against the Reference Sequence (RefSeq) genomic database.

### Mapping and expression analyses

Reads for each sample were mapped individually to the reference transcriptome using CLC Bio (default values). HTSeq (Anders *et al.* 2014) was used to quantify the number of reads mapping uniquely to each of the reference contigs.

Gene expression analysis was performed using the Bioconductor package EdgeR (v. 3.2.4.) (Robinson *et al.* 2010) in R (R Core Team 2013). Normalization factors for each sample were computed using the TMM method. We then fitted a generalized linear model (GLM) with negative binomial distribution with the terms diapause state, light cycle contrast, and diapause state * light cycle contrast (full model), and estimated dispersion using the Cox-Reid profile-adjusted likelihood (CR) method. We used GLM likelihood ratio tests and appropriate model contrasts to determine the significance of the interaction between diapause state and light cycle difference, the effect of day length on nondiapausing females, and the effect of day length on diapausing females. The *P* values from the GLM likelihood ratio tests were corrected for multiple testing using Benjamini and Hochberg’s algorithm to control for false discovery rate (FDR) (Benjamini and Hochberg 1995), with significance taken here to be <5% (FDR < 0.05).

### Functional classification

We found that most contigs blasted to related *Drosophila* species (see *Results*). In order to functionally classify these contigs, we used Gene Ontology (GO) annotation for orthologous genes in *D. melanogaster* (available from Flybase, version: FB2013_06). We used this approach instead of using GO terms obtained from the blast hits themselves due to the vastly superior GO annotation available in *D. melanogaster* (Tweedie *et al.* 2009). Significant enrichment of single GO terms was determined using a Fisher’s exact test. The *D. melanogaster* orthologs of DE genes were also analyzed using DAVID (Database for Annotation, Visualization, and Integrated Discovery) v. 6.7 (Huang *et al.* 2009a,b). DAVID clusters genes into functional groups using a “fuzzy” clustering algorithm, and then uses a Fisher’s exact test to identify significantly enriched functional groups. A functional group was considered to be significantly enriched if its enrichment score [the geometric mean (in –log scale) of the *P* values of the GO terms in the group] was >1.3 (*P* < 0.05).

### Data availability

Mapped read counts have been deposited into the Gene Expression Omnibus (GEO) under the accession code GSE76313. The transcriptome assembly has been deposited at DDBJ/EMBL/GenBank under the accession GECM00000000. The version described in this paper is the first version, GECM01000000. Raw reads have been deposited in the Sequence Read Archive (SRA) under accession codes: SRR1501259, SRR1501260, SRR1501261, SRR2910689, SRR2910692, SRR2910693, SRR2910695, SRR2910698, SRR2910701, SRR2910702, SRR2910703, and SRR2910704. Supplemental Material, Table S1 contains the GO terms associated with genes differentially expressed (DE) in response to a shorter day length in diapausing females. Table S2 contains the GO terms associated with genes DE in response to a shorter day length in nondiapausing females. Table S3 contains a list of neurogenesis/neuron development associated genes DE in response to a shorter day length in either diapausing or nondiapausing females. Table S4 contains a list of ion transport associated genes DE in response to a shorter day length in either diapausing or nondiapausing females. Table S5 contains a list of reproduction associated genes DE in response to a shorter day length in either diapausing or nondiapausing females. Table S6 contains a list of metabolic associated genes DE in response to a shorter day length in either diapausing or nondiapausing females.

## Results

### Transcriptome assembly

Using the blasting strategy described above, we obtained blast results for 99% of the contigs assembled from the RNA sequencing data (accession numbers for blast hits are available with gene expression data from the GEO, accession number: GSE76313). As expected, most of the blast hits (> 25,000 contigs) were to sequences from *D. virilis*, which is the closest relative of *D. montana* with a sequenced genome available. Almost all of the remaining hits were to other *Drosophila* species, with less than 2% (647 contigs) blasting to non-arthropod sequences. Contigs that blasted to a non-arthropod species were discarded prior to mapping. However, contigs that did not get a significant blast hit (321) were kept.

**Figure 2:**
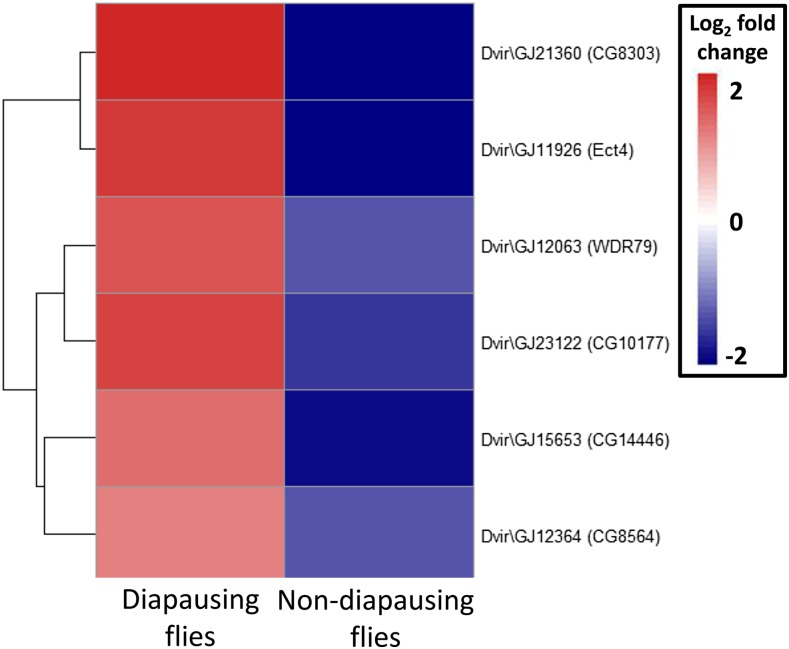
Heat map of the six contigs found to be DE in response to increasing day length in both diapausing and nondiapausing flies. Gene name is the highest significant blast hit, with *D. melanogaster* ortholog in parenthesis. DE, differentially expressed.

**Figure 3:**
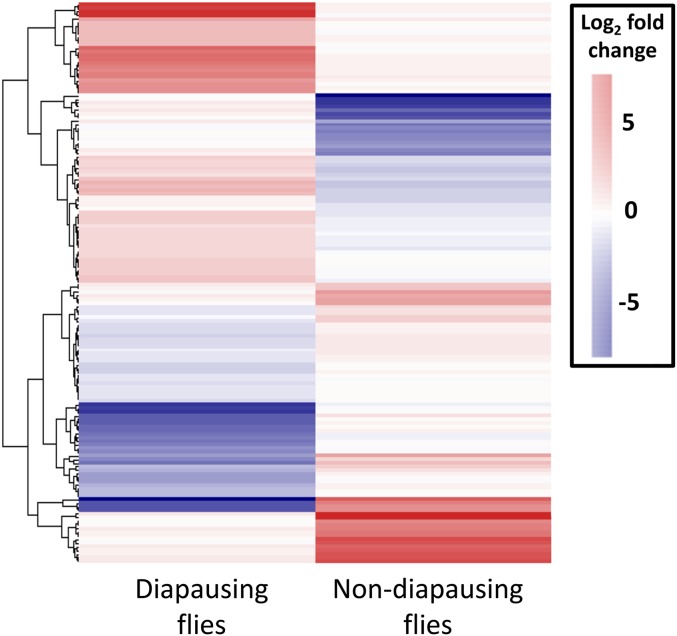
Heat map of all contigs found to be DE in response to increasing day length in diapausing and nondiapausing flies. Note the opposite patterns of expression for the majority of the contigs. DE, differentially expressed.

**Figure 4:**
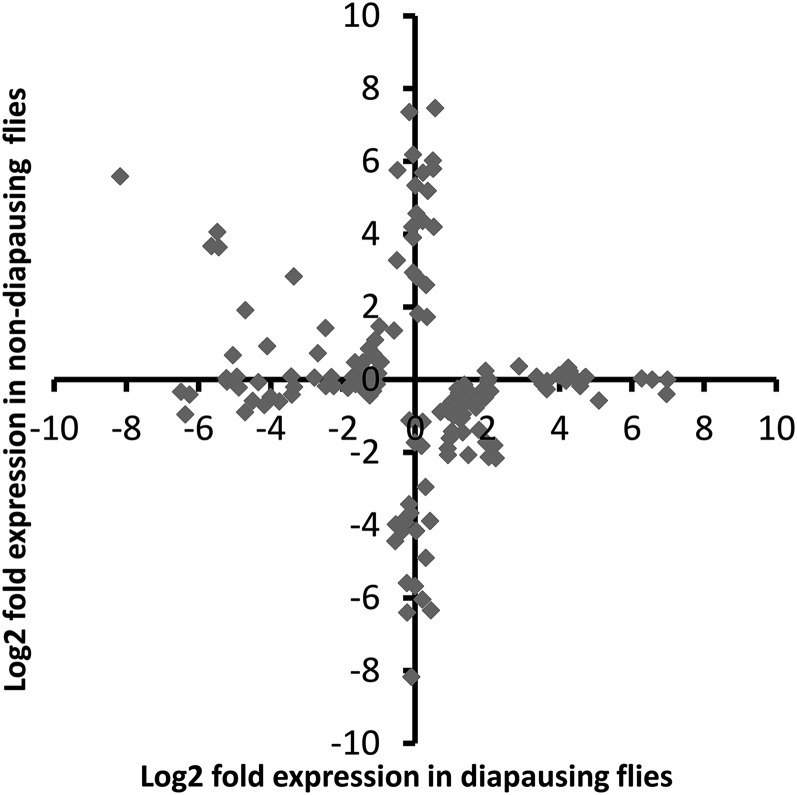
Plot of log2 fold changes for all contigs found to be DE in response to increasing day length in diapausing and nondiapausing flies. DE, differentially expressed.

Of the 69 million paired end reads obtained, approximately 47% mapped uniquely to the reference transcriptome. We found that many genes were DE as a result of maintenance under different light cycles in both diapausing and nondiapausing females. The number of genes that were DE was greater for diapausing than for nondiapausing flies (Table 1). Only six genes were found to be DE in both groups of flies, and all of these were DE in the opposite direction in the two types of fly (Figure 2), hence all showed a significant diapause state by light cycle interaction. For the rest of the DE genes in the nondiapausing and diapausing treatments, approximately 50% showed a significant diapause state by light interaction, indicating that the majority respond differently to differences in day lengths in diapausing and nondiapausing females (Figure 3). This is also supported by the finding that the correlation between DE genes from both type of flies is weakly negative (*r* = –0.21, *P* = 0.009, Figure 4).

**Table 1:** Number of contigs found to be DE in diapausing and nondiapausing females in response to a shorter day length and those showing significant diapause state by day length interaction

Group	Number of Contigs DE in Response to a Shorter Day Length	Number of Contigs Showing a Significant Diapause State by Day Length Interaction
Diapausing flies (D)	106	50
Nondiapausing flies (ND)	54	26
Common to D and ND	6	6
Total unique contigs DE across both diapause states	154	78

DE, differentially expressed; D, diapausing; ND, nondiapausing.

### Gene function

**Figure 5:**
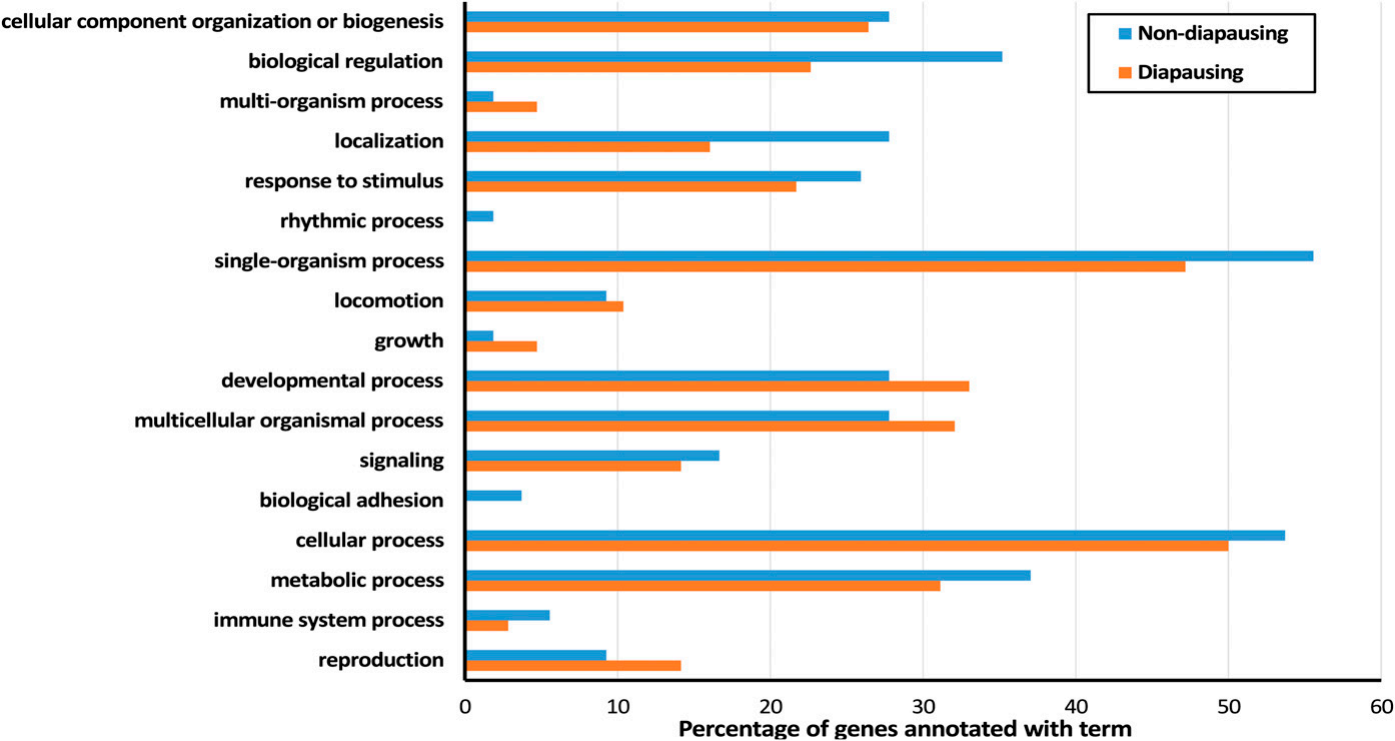
Percentage of DE genes annotated with Level 2 GO terms in nondiapausing and diapausing flies in response to a shorter day length. DE, differentially expressed; GO, gene ontology.

GO terms associated with DE genes were diverse (Figure 5, Table S1, and Table S2) with no single GO terms found to be significantly enriched in either diapausing or nondiapausing flies. However, a number of processes did stand out as potentially interesting given previous knowledge of overwintering preparation: *e.g.*, those connected to metabolic processes (Hazel 1995; Koštál *et al.* 2003) and neuron development (Montgomery and Macdonald 1990; Janssen 1992; Robertson and Money 2012). Each of these processes were associated with genes DE in both diapausing and nondiapausing flies, suggesting that there may be functional overlap even though the genes themselves differ.

### Functional clustering

Functional clustering of DE genes by DAVID found three significantly enriched clusters for diapausing flies but no significant clusters for nondiapausing flies. These clusters indicated that genes DE in response to different day lengths in diapausing flies are enriched for neurogenesis, cell migration, and fatty acid metabolism (Table 2). Although functional clusters were only identified in diapausing flies, we also found that several genes involved in the same processes were DE in nondiapausing flies, suggesting that these processes may be important for both diapausing and nondiapausing flies.

**Table 2:** Functional clustering of DE genes using DAVID produced three significantly enriched clusters for diapausing flies

Cluster	Enrichment Score	*P* Value	Description
1	1.63	0.023	Neuron projection morphogenesis, neuron development
2	1.55	0.028	Cell motion, cell migration
3	1.32	0.048	Fatty acid biosynthetic process, fatty acid metabolic process

DE, differentially expressed; DAVID, Database for Annotation, Visualization, and Integrated Discovery.

## Discussion

The onset of winter is a major challenge for insects living at high latitudes. *D. montana*, found at northern latitudes (30–70°N), has evolved a photoperiodic reproductive diapause, where short photoperiods induce flies to enter a state of dormancy prior to overwintering as adults. Diapause in *D. montana* females has been well characterized (Lumme 1978), and primarily involves the shutdown of reproduction as evidenced by much smaller ovaries in diapausing females (Salminen *et al.* 2012). The onset of diapause clearly represents a major change in physiological state (Denlinger 2002), however, the induction of diapause typically occurs over a narrow range of photoperiods. CDL is the light dark cycle when half of the females from a particular population enter diapause, but additional changes may occur both before and during diapause, at photoperiods that are shorter or longer than the CDL (Denlinger 2002), in preparation for the colder season ahead. In particular, cold tolerance has been shown to increase in both nondiapausing and diapausing flies when day length becomes shorter (Vesala *et al.* 2012b).

Here, we examined gene expression changes in response to different (shorter) day lengths using two comparisons: between summer (LD 22:2) and the CDL (LD = 18.5:5.5) (corresponding to July 4 and July 31) in nondiapausing females, and between CDL and late summer (LD = 16:8) (corresponding to July 31 and August 19) in diapausing flies preparing for overwintering. By examining differences in gene expression between flies maintained under these different light cycles, we were able to identify whether similar genes respond to a shorter day length in nondiapausing and diapausing flies.

Although we predicted that similar gene expression changes may occur in diapausing and nondiapausing females, we found that the majority of the genes that were DE in each of the comparisons differed, with only six genes shared between the two groups. These common genes showed a significant interaction between diapause state (nondiapausing or diapausing) and light cycle with genes increasing in expression in diapausing females in response to a shorter day length but decreasing in nondiapausing females (Figure 3 and Figure 4). Moreover, significant interactions between diapause state and light cycle were found for around half of the DE genes, indicating that the genes that change expression in response to differences in day lengths in the two groups of flies are largely different. This is somewhat surprising, as the magnitude of the light cycle difference and increase in cold tolerance are similar between the two comparisons (Vesala *et al.* 2012b). This suggests that additional biological processes are changing in response to differing day length and that these processes may differ between diapausing and nondiapausing females. Alternatively, although we found that the genes DE in each of the comparisons were different, it is possible that they are in fact involved in similar functional processes. To examine these ideas further, we examine the associated functional processes for the DE genes below (for a full list of processes for all the genes DE see Table S1 and Table S2).

Three of the six genes shared between the comparisons are associated with neuronal process (*CG14446*, *CG8564*, and *Ect4*) suggesting that changes to neurons are important for both types of female for preparation for a colder time ahead. Of the remaining genes, two are involved in metabolic processes [fatty acyl-CoA reductase activity (*CG8303*), and protein phosphorylation (*CG10177*)]. Since several other DE genes were also annotated with these processes (neurogenesis and metabolism), we discuss each of these processes in the context of diapause and overwintering below. The final gene shared between the comparisons (*WDR79*) is associated with nucleotide binding, suggesting that it may have a role in regulating changes in gene expression in response to different day lengths. However, future work would be needed to confirm this.

### Neurogenesis

Genes DE in response to a shorter day length in diapausing females produced a significantly enriched functional cluster for neurogenesis/neuron development, with 14% (15/106) of all the DE genes involved in these processes. Although no significant functional clusters were found in nondiapausing females, around 10% (6/54) of DE genes were also associated with neurogenesis/neuron development (Table S3). Since neuronal function is known to be particularly sensitive to changes in temperature (Montgomery and Macdonald 1990; Janssen 1992; Robertson and Money 2012) and neurons are susceptible to cold injury (Hochachka and Somero 2002), changes in this group of genes may be involved in preparatory changes to protect/adjust neuron function in anticipation of colder times ahead.

In addition, we also found genes involved in ion transport to be DE in both nondiapausing (6/54) and diapausing (4/106) flies (Table S4). Changes in these genes are likely to influence the ionic balance of cells, which may also be anticipatory for colder times, as changes in temperature are known to affect the transport mechanisms involved in the maintenance of cellular ion balance (Heitler *et al.* 1977; Kivivuori *et al.* 1990). Failure to maintain cellular ionic balance can lead to metabolic perturbations that can cause a wide range of negative consequences, particularly the loss of nerve excitation (Hochachka 1986; Koštál *et al.* 2004). Such genes may work in conjunction with neurogenesis/neuron development genes to adjust neuron physiology to enable flies to be more cold tolerant. In fact, several of these ion transport genes have been implicated in neuron-specific roles in *D. melanogaster*, including: *CG5549*, *CG14741*, *paralytic* (*para*), and *Ih*. In particular, disruptions in *para* have been shown to produce temperature-sensitive flies that exhibit a “paralytic” phenotype when exposed to a change in temperature (Suzuki *et al.* 1971; Lindsay *et al.* 2008). Intriguingly, *Ih*, which encodes for a hyperpolarization-activated cyclic nucleotide-gated channel (HCN) (Gisselmann *et al.* 2005), has been shown to be implicated in the regulation of rhythmic behaviors such as sleep (Gonzalo-Gomez *et al.* 2012), and also to affect how light signals are processed in *D. melanogaster* (Hu *et al.* 2015). As we find this gene to be downregulated in response a shorter day length in nondiapausing flies, it is possible that it is involved in cueing downstream responses to differences in light. *Ih* is also the only gene DE in either of the comparisons to be annotated as influencing circadian rhythms. This is perhaps surprising given the known influence of circadian genes on the diapause induction (Denlinger 2002; Saunders 2011). In addition, studies examining gene expression differences between nondiapausing and diapausing states typically find changes in genes involved in phototransduction or controlling circadian rhythms (Emerson *et al.* 2010; Zhao *et al.* 2015; Meuti *et al.* 2015). However, our study did not look at the onset of diapause *per se*, but rather the changes taking place prior to and after the occurrence of diapause and, thus, it appears that circadian genes have a more limited role in these comparisons (also see Salminen *et al.* 2015).

### Reproduction

Both diapausing and nondiapausing flies show DE genes annotated with the GO terms “reproductive process” or “reproduction,” with 14% (15/106) of DE genes in diapausing flies and 9% (5/54) in nondiapausing flies (Table S5). Since we only made comparisons within reproductive states (diapausing and nondiapausing) this finding suggests that females begin altering their reproductive state in response to a shorter day length both prior to and after entering diapause. For nondiapausing flies, three genes involved in gonad development (*thr*, *wb*, and *fz2*) were DE, with a fourth gene involved in oogenesis (*asun*) found to be strongly downregulated. In diapausing females, we also observed that several genes involved in oogenesis (*wek*, *Gprk2*, and *wash*) and two yolk protein genes (*Yp2* and *Yp3*) were downregulated, suggesting that there is additional shutdown of reproductive processes during diapause. Taken together, these results suggest that females use day length to cue changes in their reproductive states both before and after entering into diapause.

Interestingly, Zhao *et al.* (2015) found that *Yp1*, *Yp2*, and *Yp3* were strongly DE between diapause states in the heads of *D. melanogaster*. Although the function of these genes in the head is unknown, previous work has shown that these genes are also involved in neurogenesis (Neumüller *et al.* 2011). This suggests that the DE of *Yp2* and *Yp3* in our study may be responsible for changes in neurons (as discussed above) rather than, or in addition to, reproduction. Future work examining the expression of *Yp2* and *Yp3* in different tissues of *D. montana* will allow us to tease apart these, or other, potential roles.

### Metabolic processes

**Figure 6:**
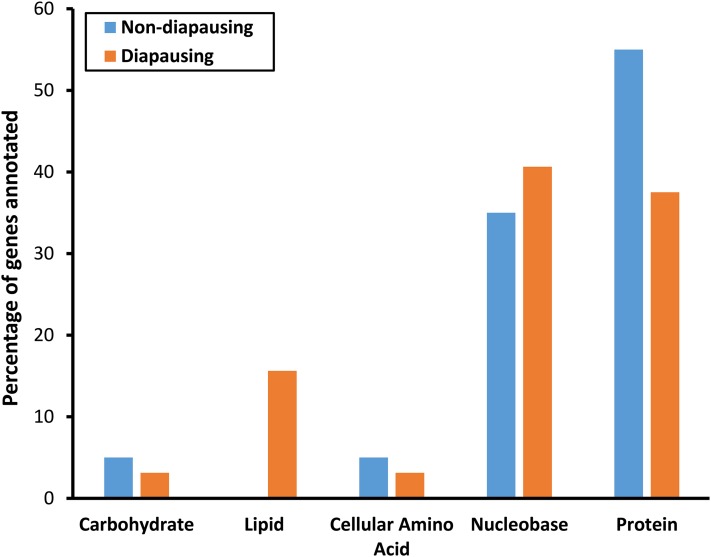
Percentage of genes annotated for each of the primary metabolic processes for nondiapausing and diapausing flies.

An increase in cold tolerance is known to involve a shift in the metabolic profile, as well as the production of, cryoprotectants, which act to maintain the osmotic balance and stabilize the membrane structures of a cell (Hazel 1995; Koštál *et al.* 2003). Since cold tolerance is known to increase as photoperiod decreases in *D. montana* (Vesala *et al.* 2012b), we expected to find genes involved in metabolic processes to be DE in response to reduced photoperiod in both types of flies. Consistent with this, we found that around 30% of genes DE in response to a shorter day length in nondiapausing (17/54) and diapausing flies (31/106) were involved in primary metabolic processes (Table S6). Splitting the primary metabolic GO term into its constitute parts showed that the majority of the metabolic genes were involved in nucleobase and protein metabolism, with similar proportions of each term found for both comparisons (Figure 6).

It is unclear what potential role changes in nucleobase metabolism have in our study, but they are likely to influence changes in the transcription of particular subsets of genes. Moreover, although it is not clear which genes/processes are regulated by the majority of the nucleobase metabolism genes DE in our study, many are involved in controlling development including gonadal and embryonic processes. These changes likely reflect the modifications in reproductive status as discussed above, rather than nucleobase metabolism playing a role in overwintering preparation *per se*.

Changes in protein metabolism (particularly changes in amino acid concentrations in the hemolymph) have previously been associated with diapause in a number of insect species including: *Ostrinia sp*. (Lepidoptera) (Morgan and Chippendale 1983), *Leptinotarsa decemlineata* (Coleoptera) (Lefevere *et al.* 1989), and *Pyrrhocoris apterus* (Hemiptera) (Koštál *et al.* 2011). Such changes are likely to have two roles in insect diapause: increasing cold tolerance (see Lee 1991), and serving as nutrient storage (Hahn and Denlinger 2011). Storage of amino acids is thought to be important for diapause in order for the organism to survive the dormancy period and to allow for the remodeling of tissues with the onset of spring (Hahn and Denlinger 2011). Several studies have shown that insects often store amino acids as hexameric storage proteins, and in *D. melanogaster* the major storage proteins are the hexameric larval serum proteins *Lsp1* and *Lsp2* (Haunerland 1996). Interestingly, we found that the gene coding for one of these, *Lsp2*, is upregulated in response to reduced day length in nondiapausing flies, suggesting that females use this gene to store amino acids in preparation for seasonal change. This gene has been previously associated with storage of amino acids during diapause in several insect orders [*e.g.*, Coleoptera (Koopmanschap *et al.* 1992) and Lepidoptera (Godlewski *et al.* 2001)]. Since diapause is likely to have evolved independently in numerous insect taxa (for reviews see: Denlinger 2002; Saunders 2011), this suggests that *Lsp2* has been coopted into storing amino acids during winter numerous times.

Previous work examining insect diapause has consistently shown large expression changes in genes involved in lipid metabolism (Sim and Denlinger 2009; Kubrak *et al.* 2014; Salminen *et al.* 2015; Zhao *et al.* 2015). In this study, we only found five genes (*CG16904*, *ifc*, *Acetyl-CoA carboxylase*, *Yp2*, and *Yp3*) DE in response to a shorter day length in diapausing flies (see Table S6). This finding suggests that changes in lipid metabolism, though important in the transition between nondiapause and diapause stages, vary only a little in response to differences in day length.

We suggest that the changes we observed in metabolism are likely be involved in altering *D. montana’s* cold tolerance and/or nutrient storage, but it is also possible that such changes are working in concert with, or are a result of, changes in feeding activity. It is known that several species of *Drosophila* reduce their food intake when diapausing (Matsunaga *et al.* 1995; Kubrak *et al.* 2014) and thus it is possible that *D. montana* alter the amount of food they consume in response to a change in day length. For instance, flies may increase their feeding activity at shorter day lengths, in preparation for diapause and overwintering when feeding will be reduced. Such a change in feeding may thereby cause a difference in the expression of genes involved in food storage (such as *Lsp2*, above). To confirm this hypothesis, future work examining how day length alters feeding activity and how such changes may impact the expression of metabolism genes, how subsequent metabolites are assimilated, and how they may affect cold tolerance/overwintering survival are needed.

### Relation of reduced day length to cold acclimation

Both the shortening of the photoperiod and decreased temperature are known to lead to increased cold tolerance in *D. montana* (Vesala *et al.* 2012b). As a result, we can ask if DE genes in response to cold are the same as those DE in response to a shorter day length. In our previous study, we examined gene expression differences in response to cold (rather than light) in *D. montana* (Parker *et al.* 2015), but we find that only two genes, *Lsp2* and *CG6084*, overlap with the DE genes in this study. Both of these genes were found to be DE in the nondiapausing comparison and *Lsp2* (which, as discussed above, is a storage protein), was found to be downregulated during cold acclimation (rather than upregulated in response to a shorter day length). The reason for the downregulation of this gene is unknown, but may reflect the fact that cold acclimation is energetically costly. *CG6084*, on the other hand, is reported as having aldo/keto reductase activity and an oxidoreductase domain, but the processes it is involved in are otherwise unknown. Given that it is downregulated both in response to a shorter day length and the onset of cold (cold acclimation), we hypothesize that it may have an important role in increasing cold tolerance.

Overall, there is very little overlap between DE genes during cold acclimation and those associated with decreasing day length. This is somewhat surprising as both nondiapausing and diapausing flies show an increase in cold tolerance (Vesala *et al.* 2012a,b), leading to the question of why do we not see a greater proportion of shared genes? One reason may simply be that, since an increase in cold tolerance can be achieved by production of many different metabolites, those produced may be specific to each environmental cue. Cold acclimation, diapause, and overwintering preparation are all complex induced phenotypes, which are energetically costly. As such, the ability and cost to produce specific metabolites may be different in each situation, resulting in the utilization of different pathways to increase cold tolerance. Future work, examining the shift in metabolic profiles during different phases of cold acclimation and diapause, is needed to examine this further.

### Future work

In our study, we identified genes that changed in expression between specific day lengths. We interpret these changes as consistent with preparing for seasonal changes but, due the nature of our experiment, we have not shown whether such genes have constant patterns of expression over all of the day lengths that are naturally experienced by *D. montana* as they prepare for winter. Thus, future work examining the expression of the candidate genes we have identified here across a more continuous range of day lengths would be particularly interesting. In this study, we also suggest functional roles for several of the genes identified as being DE. However, due to the correlative nature of RNA-seq, future functional genetic work using RNAi or CRISPR to experimentally alter the expression of such candidate genes is now needed to test such suggestions.

## Supplementary Material

Supplemental Material
