# Supplementary material for: Preparing for Winter: The Transcriptomic Response Associated with Different Day Lengths in Drosophila montana
Source: G3 (Bethesda). 2016 Mar 11;6(5):1373–81. doi: 10.1534/g3.116.027870 (PMC4856088; doi:10.1534/g3.116.027870)
Supplement: Supplemental Material [file supp_6_5_1373__index.html]

Preparing for Winter: The Transcriptomic Response Associated with Different Day Lengths in Drosophila montana — Supplemental Material 

# Preparing for Winter: The Transcriptomic Response Associated with Different Day Lengths in *Drosophila montana*

## Supplemental Material for Parker, Ritchie, and Kankare, 2016

**Files in this Data Supplement:**

- Table S1 - GO terms associated with genes DE in response to a shorter day length in diapausing females. (.xlsx, 48 KB)
- Table S2 - GO terms associated with genes DE in response to a shorter day length in non-diapausing females. (.xlsx, 35 KB)
- Table S3 - Neurogenesis / neuron development associated genes DE in response to a shorter day length in either diapausing or non-diapausing females. (.xlsx, 14 KB)
- Table S4 - Ion transport associated genes DE in response to a shorter day length in either diapausing or non-diapausing females. (.xlsx, 13 KB)
- Table S5 - Reproduction associated genes DE in response to a shorter day length in either diapausing or non-diapausing females. (.xlsx, 16 KB)
- Table S6 - Metabolic associated genes DE in response to a shorter day length in either diapausing or non-diapausing females. (.xlsx, 19 KB)
